# *Plasmodium vivax* but Not *Plasmodium falciparum* Blood-Stage Infection in Humans Is Associated with the Expansion of a CD8^+^ T Cell Population with Cytotoxic Potential

**DOI:** 10.1371/journal.pntd.0005031

**Published:** 2016-12-08

**Authors:** Julie G. Burel, Simon H. Apte, James S. McCarthy, Denise L. Doolan

**Affiliations:** 1 Molecular Vaccinology Laboratory, QIMR Berghofer Medical Research Institute, Brisbane, Australia; 2 School of Medicine, The University of Queensland, Brisbane, Australia; 3 Clinical Tropical Medicine Laboratory, QIMR Berghofer Medical Research Institute, Brisbane, Australia; 4 Australian Institute of Tropical Health and Medicine, James Cook University, Cairns, Australia; 5 Centre for Biosecurity and Tropical Infectious Disease, James Cook University, Cairns, Australia; University of California San Diego School of Medicine, UNITED STATES

## Abstract

*P*. *vivax* and *P*. *falciparum* parasites display different tropism for host cells and induce very different clinical symptoms and pathology, suggesting that the immune responses required for protection may differ between these two species. However, no study has qualitatively compared the immune responses to *P*. *falciparum* or *P*. *vivax* in humans following primary exposure and infection. Here, we show that the two species differ in terms of the cellular immune responses elicited following primary infection. Specifically, *P*. *vivax* induced the expansion of a subset of CD8^+^ T cells expressing the activation marker CD38, whereas *P*. *falciparum* induced the expansion of CD38^+^ CD4^+^ T cells. The CD38^+^ CD8^+^ T cell population that expanded following *P*. *vivax* infection displayed greater cytotoxic potential compared to CD38^-^ CD8^+^ T cells, and compared to CD38^+^ CD8^+^ T cells circulating during *P*. *falciparum* infection. We hypothesize that *P*. *vivax* infection leads to a stronger CD38^+^ CD8^+^ T cell activation because of its preferred tropism for MHC-I-expressing reticulocytes that, unlike mature red blood cells, can present antigen directly to CD8^+^ T cells. This study provides the first line of evidence to suggest an effector role for CD8^+^ T cells in *P*. *vivax* blood-stage immunity. It is also the first report of species-specific differences in the subset of T cells that are expanded following primary *Plasmodium* infection, suggesting that malaria vaccine development may require optimization according to the target parasite.

**Trial Registration:**

anzctr.org.au ACTRN12612000814875; anzctr.org.au ACTRN12613000565741; anzctr.org.au ACTRN12613001040752; ClinicalTrials.gov NCT02281344; anzctr.org.au ACTRN12612001096842; anzctr.org.au ACTRN12613001008718

## Author Summary

The specific immune responses that contribute to protective immunity in humans following *Plasmodium* infection are yet to be fully characterized. The species *P*. *vivax* and *P*. *falciparum* account for most human infections, yet little is known about *P*. *vivax* specific immune responses and whether they are similar to or distinct from *P*. *falciparum*. Here, we establish that *P*. *vivax* and *P*. *falciparum* elicit distinct cellular immune responses following primary infection, with the expansion of a subset of CD38^+^ CD8^+^ T cells with a cytotoxic potential in *P*. *vivax* but not in *P*. *falciparum* infection. This study provides the first evidence for the activation of CD8^+^ T cells in *P*. *vivax* blood-stage infection and demonstrates the existence of species-dependent host immune responses to malaria. These findings have important implications for *P*. *vivax* vaccine development, and suggest that future malaria vaccine studies should be adapted according to the target *Plasmodium* spp.

## Introduction

Malaria vaccine research efforts have been directed predominantly at *P*. *falciparum*, since globally it is the major cause of malaria-related mortality [1]. However, it is now recognized that *P*. *vivax* is poised to become the dominant species in areas where it is endemic [2] and can be associated with severe pathology [2,3]. Yet, compared to what is known about responses to *P*. *falciparum*, little is known about immune responses to *P*. *vivax* infection. This lack in knowledge is due in part to confounders that are present in samples from naturally-infected individuals living in malaria-endemic regions where parasitic co-infections and cross-species immunity are present; and technical difficulties associated with experimental infection of humans due to a lack of a method for the continuous *in vitro* culture of *P*. *vivax* [4]. It has been generally assumed that *P*. *vivax* would elicit similar immune responses compared to *P*. *falciparum*. However, the two parasites display very different features in terms of life cycle, invasion mechanism and immunopathology [2,3,5] and thus may generate distinct host specific immune responses. A few studies have compared global frequencies of circulating lymphocyte populations during *P*. *falciparum* or *P*. *vivax* infection in naturally infected humans [6,7], but have not investigated their activated or effector phenotype.

The recent establishment of different models of Controlled Human Malaria Infection (CHMI) provides the opportunity to obtain samples from malaria-naive healthy volunteers following first exposure to *Plasmodium* blood-stage parasites, thereby greatly enhancing our understanding of the host-parasite immune response [8,9]. Until recently, such experimental infection studies could be done only with *P*. *falciparum* due to the lack of a continuous *in vitro* culture system of *P*. *vivax* as a source of parasitized red blood cells [8]. Recently, however, a cell bank of cryopreserved *P*. *vivax* infected erythrocytes was successfully derived from a naturally-infected individual and used to experimentally infect malaria-naive healthy adult volunteers, establishing for the first time a CHMI model with *P*. *vivax* [10].

Here, we have taken advantage of this novel resource to compare cellular immune responses generated following experimental blood-stage infection of naive volunteers with *P*. *vivax* or *P*. *falciparum*. Overall, we found marked differences in the immune profiles generated following infection with the two species. Specifically, *P*. *vivax* but not *P*. *falciparum* infection led to the expansion of a specific subset of CD8^+^ T cells which were associated with an activated phenotype and cytotoxic potential. This study enhances our understanding of *P*. *vivax* associated immunity and *Plasmodium* species-specific immunity, identifying for the first time components of the immune response to blood-stage infection that are species-specific.

## Methods

### Ethics

Experimental infection of malaria-naive healthy adult volunteers was undertaken at QPharm Pty Ltd (Brisbane, Australia); all clinical studies were registered on the Australian and New Zealand Clinical Trials Registry (ANZCTR): *P*. *falciparum* clinical trial ID numbers ACTRN12612000814875, ACTRN12613000565741, ACTRN12613001040752 and NCT02281344; and *P*. *vivax* clinical trial numbers ACTRN12612001096842 and ACTRN12613001008718, with written informed consent and approval of the QIMR Berghofer Medical Research Institute Human Research Ethics Committee (QIMRB-HREC) and the Western Institutional Review Board (ethics board for the trial sponsor, Program for Appropriate Technology in Health, PATH).

### Sample collection and processing

Inoculum preparation, volunteer recruitment, infection, monitoring and treatment were performed as described previously for *P*. *falciparum* [11] or *P*. *vivax* [10]. In brief, healthy malaria-naive individuals were intravenously inoculated with freshly thawed *P*. *falciparum* 3D7 or *P*. *vivax* parasitized erythrocytes and treated with anti-malarial drugs when the parasitemia exceeded the approximate threshold of 10,000 parasites/mL, at day 7–8 post-infection or day 14 post-infection for *P*. *falciparum* or *P*. *vivax*, respectively. The infecting dose for *P*. *falciparum* was 1,800 viable parasitized red blood cells. Parasite growth modeling using *in silico* analysis estimated that the infecting dose for *P*. *vivax* was 15 fold lower compared to *P*. *falciparum* (*Khoury D & McCarthy JS*, *in preparation*). Blood samples were collected prior to infection, at day 7 post-infection for *P*. *falciparum* infected volunteers, and day 14 post-infection for *P*. *vivax* infected volunteers. Peripheral blood collected in Lithium Heparin Vacutainers (Becton Dickinson) was either used directly for flow cytometry analysis, or peripheral blood mononuclear cells (PBMC) isolated using standard Ficoll density gradient centrifugation.

### Determination of parasitemia and kinetics

Parasitemia was determined using a consensus *P*. *falciparum* or *P*. *vivax* species-specific quantitative PCR assay as previously described [12]. Parasite levels were assessed once daily until day four post-infection and then twice daily until treatment. All samples were batch tested in triplicate together after each study completion. Limit of detection was 64 parasites/ml [12]. Exponential growth equation fitting parasitemia kinetics for *P*. *falciparum* or *P*. *vivax* infected volunteers was calculated with GraphPad Prism (version 6.0).

### *Ex vivo* phenotyping of lymphocytes by flow cytometry on whole blood

Staining buffer was PBS supplemented with 0.5% FCS and 4 mM EDTA. Whole blood collected in Lithium Heparin vacutainers was lysed and fixed with BD FACS lysing solution (Becton Dickinson) and lymphocytes permeabilized with BD FACS permeabilising solution 2 (Becton Dickinson) according to the manufacturer’s instructions. Cells were then resuspended in 50 μl of staining buffer containing anti-human CD4-BV510 (Becton Dickinson, 1:200 dilution), anti-human CD8-APC-H7 (Becton Dickinson, 1:400 dilution), anti-human CD19-PE-Cy7 (Biolegend, 1:200 dilution), anti-human CD38-APC (Biolegend, 1:400 dilution), anti-human Perforin-PE (Biolegend, 1:400 dilution), anti-human Granzyme B-Pacific Blue (Biolegend, 1:400 dilution) and 1 μl of human Fc receptor blocking solution (Human TruStain FcX, Biolegend) for 30 minutes at room temperature, washed and resuspended in staining buffer before acquisition on LSR Fortessa 4 (Becton Dickinson) with Diva software. FlowJo software version 6.0 was used for gating.

### Cell sorting

CD38^+^ CD8^+^ T cells, CD38^-^ CD8^+^ T cells as well as CD8^-^ cells were sorted from freshly isolated PBMC. Approximately 10x10^6^ cells were resuspended in 50 μl of staining buffer containing anti-human CD4-BV510 (Biolegend, 1:200 dilution), anti-human CD8-APC-H7 (Becton Dickinson, 1:400 dilution) and anti-human CD38-PerCpCy5.5 (Biolegend, 1:400 dilution) for 20 minutes at 4°C, washed and resuspended in staining buffer. Just before the sorting, 1 μg/mL of propidium iodide (Sigma-Aldrich) was added to allow for assessment of viability. Pi^-^CD8^+^CD4^-^CD38^+^, Pi^-^CD8^+^CD4^-^CD38^-^, and Pi^-^CD8^-^ cells were sorted using a BD Aria III cell sorter (Becton Dickinson) directly in staining buffer and kept on ice until further use for *in vitro* assays.

### CD107a-based degranulation assay

Sorted CD38^+^ and CD38^-^ CD8^+^ T cells were plated at 50,000 cells/well in RPMI 1640 containing 25 mM Hepes, 2 mM L-glutamine (Invitrogen), and supplemented with 10 units/mL of Penicillin (Life Technologies), 10 μg/mL of Streptomycin (Life Technologies) and 10% fetal bovine serum (Life Technologies) in a 96-well plate pre-coated overnight with 10 μg/mL of anti-human CD3 OKT3 antibody (Biolegend) together with 0.75x10^6^ cells/mL autologous CD8^-^ cells, anti-human CD107a-FITC (Biolegend, 1:200 dilution) and 1 μg/mL of co-stimulatory antibodies anti-human CD28 and anti-human CD49d (Becton Dickinson) for 5 hours at 37°C in an atmosphere of 5% C0_2_. Following stimulation, cells were resuspended in 20 μl of staining buffer containing anti-human CD4-BV510 (Biolegend, 1:200 dilution), anti-human CD8-APC-H7 (Becton Dickinson, 1:400 dilution) for 20 minutes at 4°C, washed and resuspended in staining buffer before acquisition on LSR Fortessa 4 (Becton Dickinson) with Diva software. FlowJo version 6.0 was used for gating.

## Results

### *P*. *falciparum* and *P*. *vivax* primary blood-stage infection in humans generated similar parasitemia curves

*P*. *falciparum* and *P*. *vivax* experimental infection of malaria-naive volunteers was performed under similar procedures [10,11]. However, due to logistical reasons associated with parasite density in the inoculum stock, and a lack of a continuous *in vitro* culture system for *P*. *vivax* [4], the infecting dose for *P*. *vivax* was estimated to be 15-fold lower than that used in the *P*. *falciparum* studies (*Khoury D & McCarthy JS*, *in preparation)*. Demographics of *P*. *falciparum* and *P*. *vivax* infected volunteers were comparable in term of age, gender, BMI and ethnicity (**S1 Table**).

**Fig 1:**
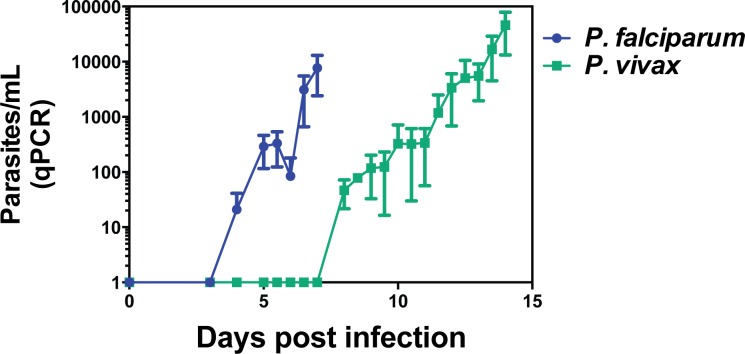
Kinetics of parasite levels during experimental blood-stage infection with *P*. *falciparum* or *P*. *vivax*. Malaria-naive volunteers were infected intravenously at day 0 with freshly thawed cryopreserved *P*. *falciparum* or *P*. *vivax* infected erythrocytes. Parasite growth modeling using *in silico* analysis estimated that the *P*. *vivax* infecting dose was 15 times lower compared to the *P*. *falciparum* infecting dose (*Khoury D & McCarthy JS*, *in preparation)*. Parasite levels in peripheral blood of infected volunteers were determined using consensus *Plasmodium* species-specific qPCR as previously described [12]. Blood samples used to assess immune responses were collected prior to infection. Graphs show mean data from 19 volunteers from three independent cohorts (*P*. *falciparum*) and 8 volunteers from four independent cohorts (*P*. *vivax*); error bars indicate SD.

Parasitemia growth curves determined by quantitative PCR (qPCR) from *P*. *falciparum* and *P*. *vivax* experimental infection studies were similar for both parasites (**Fig 1** and **Table 1**) except for the delayed onset of detectable blood-stage parasitemia with *P*. *vivax*. Specifically, *P*. *falciparum* parasites were detected as early as day 4 of infection whereas *P*. *vivax* parasites were detected at day 8 of infection, consistent with the differences in the size of the starting inocula. Interestingly, all individuals infected with *P*. *vivax* developed symptoms of malaria before the time of treatment while more than 40% of *P*. *falciparum* infected individuals were asymptomatic until anti-malarial drug administration (**S2 Table**).

**Table 1:** Statistics of exponential growth equation fitting the parasitemia curves of *P*. *falciparum* and *P*. *vivax* infected naïve volunteers.

	*P*. *falciparum*	*P*. *vivax*
**Growth rate** (days^-1^)	2.2 [1.5–2.9]	1.9 [1.3–2.5]
**Doubling time** (days)	0.31 [0.24–0.46]	0.36 [0.27–0.54]
**R square**	0.60	0.63

### *P*. *falciparum* and *P*. *vivax* primary blood-stage infection in humans elicited qualitatively different immune responses

**Fig 2:**
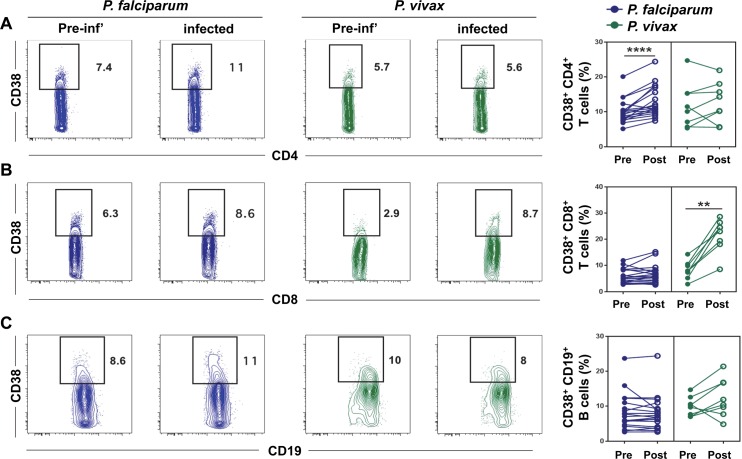
The frequency of CD38^+^ T cells and B cells circulating during experimental blood-stage infection with *P*. *falciparum* or *P*. *vivax*. Blood samples were collected from malaria-naive volunteers prior to (pre) and post-infection (post) with *P*. *falciparum* or *P*. *vivax* infected erythrocytes, as described in legend to Fig 1. The frequency of CD38^+^ cells was determined by flow cytometry in (A) CD4^+^ T cells, (B) CD8^+^ T cells, or (C) CD19^+^ B cells. Differences in cell frequencies between pre- and post-infection were determined using the non-parametric paired Wilcoxon test. Plots show representative staining of CD38 from one *P*. *falciparum* infected volunteer and one *P*. *vivax* infected volunteer. Graphs show paired data from 19 volunteers from three independent cohorts (*P*. *falciparum*) and 8 volunteers from four independent cohorts (*P*. *vivax*); ****, *p* < 0.0001; **, *p* < 0.01.

In order to compare cellular immune responses to infection between *P*. *falciparum* and *P*. *vivax* infected volunteers, we determined the phenotype of lymphocytes *ex vivo* from whole blood samples obtained prior to infection and during infection once the parasitemia exceeded 10,000 parasites/mL (which corresponded to day 7 and day 14 for *P*. *falciparum* and *P*. *vivax* infected volunteers, respectively). CD38 is a surface glycoprotein that modulates cell adhesion, signal transduction and intracellular Ca^2+^ levels, and is specifically upregulated on lymphocytes following activation [13]. We have recently shown that the frequency of CD38^+^ T cells and B cells circulating in the peripheral blood of test volunteers was dynamically regulated during experimental blood-stage infection with *P*. *falciparum* and that the expansion of CD38^+^ CD4^+^ T cells following infection was inversely correlated with parasite burden [14]. Thus, we compared the frequencies of CD38^+^ T cells and B cells circulating before and after infection in *P*. *falciparum* or *P*. *vivax* infected volunteers. There was a higher frequency of CD38^+^ CD4^+^ T cells circulating following infection in *P*. *falciparum* but not *P*. *vivax* infected volunteers (**Fig 2A**). Conversely, *P*. *vivax* infection but not *P*. *falciparum* elicited a higher frequency of circulating CD38^+^ CD8^+^ T cells (**Fig 2B**). No significant differences were observed in the frequency of CD38^+^ B cells circulating following infection with *P*. *falciparum* or *P*. *vivax* (**Fig 2C**). There was no correlation between the expansion of CD38^+^ CD8^+^ T cells following infection and parasite burden (**S1 Fig**). Overall, these data suggest that the quality of the immune response generated following primary blood-stage infection in humans is *Plasmodium* species-dependent.

### CD38^+^ CD8^+^ T cells expanded in *P*. *vivax* infection are associated with a cytotoxic function

**Fig 3:**
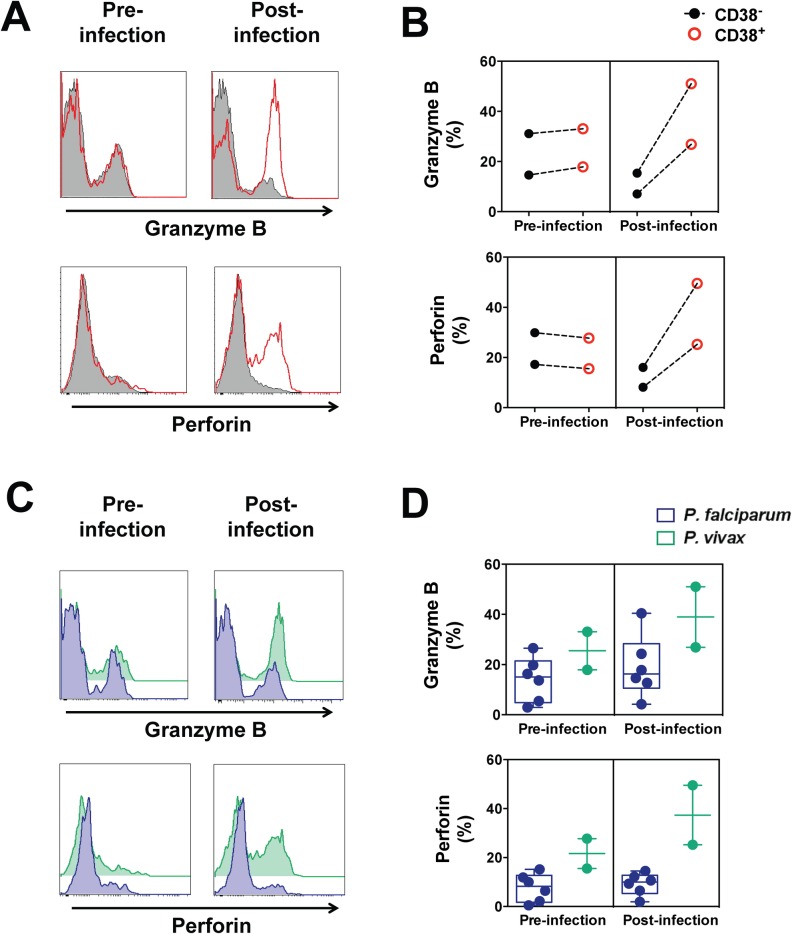
Granzyme B and perforin expression in circulating CD38^+^ CD8^+^ T cells following *P*. *vivax* blood-stage infection. Blood samples were collected from malaria-naive volunteers prior to and post-infection with *P*. *falciparum* or *P*. *vivax* infected erythrocytes, as described in legend to Fig 1. The expression of granzyme B and perforin within CD38^+^ CD8^+^ T cells and CD38^-^ CD8^+^ T cells was determined by flow cytometry. (A) Representative staining of granzyme B and perforin in CD38^+^ and CD38^-^ CD8^+^ T cells from one *P*. *vivax* infected volunteer. (B) The frequency of cells expressing granzyme B and perforin in CD38^+^ or CD38^-^ CD8^+^ T cells circulating pre- and post-infection with *P*. *vivax*. (C) Representative staining of granzyme B and perforin in circulating CD38^+^ CD8^+^ T cells from one *P*. *falciparum* infected volunteer and one *P*. *vivax* infected volunteer. (D) The frequency of cells expressing granzyme B and perforin in circulating CD38^+^ CD8^+^ T cells pre- or post-infection in *P*. *vivax* or *P*. *falciparum* infected volunteers. Graphs show mean data from 6 volunteers (*P*. *falciparum*) or 2 volunteers (*P*. *vivax*); error bars indicate SD.

Since very little information on *P*. *vivax* protective immune responses is available, we aimed to further understand the contribution of CD38^+^ CD8^+^ T cells to *P*. *vivax* blood-stage immunity. Effector CD8^+^ T cells perform classical cytotoxic functions by killing infected cells through perforin-mediated dependent mechanisms. To determine the cytotoxic potential of CD38^+^ CD8^+^ T cells generated during *P*. *vivax* infection, we measured their intracellular expression of granzyme B and perforin by flow cytometry, in an independent cohort (n = 2 because of logistics associated with vivax experimental infection). We found similar expression of granzyme B and perforin in CD38^+^ and CD38^-^ CD8^+^ T cells before infection (**Fig 3A and 3B**). However, post-infection, CD38^+^ CD8^+^ T cells had a greater expression of granzyme B and perforin compared to CD38^-^ CD8^+^ T cells (**Fig 3A and 3B**). Additionally, CD38^+^ CD8^+^ T cells circulating during infection contained a higher amount of granzyme B and perforin in *P*. *vivax* infected volunteers compared to *P*. *falciparum* infected volunteers (**Fig 3C and 3D**) whereas no differences were observed prior to infection.

**Fig 4:**
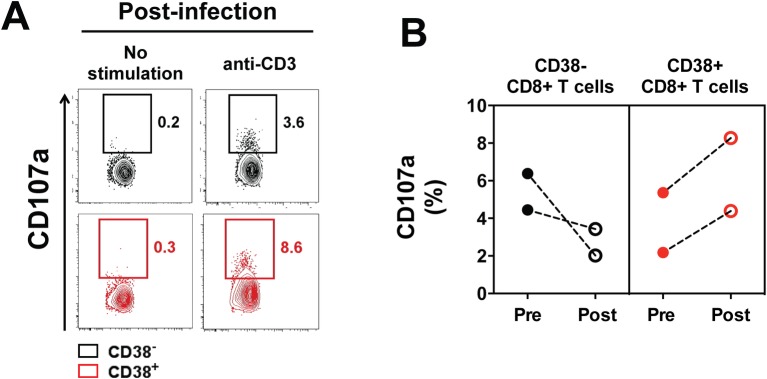
Degranulation in circulating CD38^+^ and CD38^-^ CD8^+^ T cells following *P*. *vivax* blood-stage infection. Blood samples were collected from malaria-naive volunteers prior to and post-infection with *P*. *falciparum* or *P*. *vivax* infected erythrocytes, as described in legend to Fig 1. Degranulation of CD38^+^ and CD38^-^ CD8^+^ T cells was determined by flow cytometry using a CD107a-based degranulation assay on sorted cells after five hours stimulation with plate-bound anti-human CD3 (10 μg/mL). (A) Representative staining of CD107a in circulating CD38^+^ and CD38^-^ CD8^+^ T cells from one *P*. *vivax* infected volunteer. (B) Frequency of CD107a^+^ cells after stimulation (background subtracted) in circulating CD38^+^ and CD38^-^ CD8^+^ T cells pre- or post-infection in *P*. *vivax* infected volunteers. Graphs show mean data from 2 volunteers.

In order to further investigate the cytotoxic potential of CD38^+^ CD8^+^ T cells and CD38^-^ CD8^+^ T cells circulating during *P*. *vivax* infection, we tested their ability to degranulate *in vitro* following TCR stimulation. Prior to infection, CD38^+^ and CD38^-^ CD8^+^ T cells had the same ability to degranulate, whereas post-infection CD38^+^ CD8^+^ T cells had a higher degranulation compared to CD38^-^ CD8^+^ T cells (**Fig 4**).

Overall, these findings suggest that CD38^+^ CD8^+^ T cells have a greater cytotoxic potential compared to CD38^-^ CD8^+^ T cells and that this cytotoxic function is specifically activated in the CD38^+^ CD8^+^ T cells circulating during *P*. *vivax* infection but not *P*. *falciparum* infection.

## Discussion

Herein we report on the first study to compare the quality of cellular immune responses elicited in humans by experimental blood-stage infection with *P*. *vivax* or *P*. *falciparum*. In this study we utilized a model of controlled infection of malaria-naive human volunteers, thereby avoiding potential confounders of pre-existing immunity and cross-species immunity. We found marked differences between responses to the two *Plasmodium* species in terms of the phenotype of T cells that expanded during infection. *P*. *falciparum* infection elicited a significant expansion of CD38^+^ CD4^+^ T cells whereas *P*. *vivax* infection led to the expansion of CD38^+^ CD8^+^ T cells. We have recently shown that the frequency of circulating CD38^+^ CD4^+^ T cells was significantly increased following experimental infection with *P*. *falciparum* and inversely correlated with parasite levels [14]. Here we show that *P*. *vivax* blood-stage infection elicited a substantially different type of immune response compared to *P*. *falciparum*, with significant changes in the CD8^+^ T cell compartment rather than in the CD4^+^ T cell compartment. There was no significant association between the expansion of CD38^+^ CD8^+^ T cells and parasite burden in *P*. *vivax* infected volunteers, suggesting that the expansion of CD38^+^ CD4^+^ T cells in *P*. *falciparum* infection and the expansion of CD38^+^ CD8^+^ T cells in *P*. *vivax* infection might have distinct contributions to the immune response to blood-stage infection.

A possible explanation for the qualitative differences observed between *P*. *falciparum* and *P*. *vivax* associated immune responses may relate to their distinct tropism during blood-stage infection. *P*. *vivax* merozoites preferentially invade reticulocytes [15] which, although they are enucleated, still express MHC-I molecules which remain from the *nucleated* reticuloblast stage. This is in contrast to mature erythrocytes that have completely lost expression of MHC molecules. Previous work using a genetically engineered mouse model of murine malaria has shown that CD8^+^ T cells can be activated by parasitized erythroblasts but not parasitized mature red blood cells through MHC-I dependent mechanisms [16]. Thus, we hypothesize that the higher proportion of infected reticulocytes in *P*. *vivax* infection leads to the activation of a higher proportion of CD38^+^ CD8^+^ T cells in an MHC-I dependent manner in comparison to *P*. *falciparum* infection.

While it is established that CD4^+^ T cells and parasite-specific antibodies are critical for protective immune responses to blood-stage malaria [17], the contribution of CD8^+^ T cells is less clear. Studies in mice have shown that CD8^+^ T cells were activated and associated with protective function in lethal [18] or chronic [19] blood-stage malaria. However, no association between CD8^+^ T cells and protective immunity to primary blood-stage infection in humans has been reported yet. Our data suggest that the CD38^+^ CD8^+^ T cells that specifically expand during *P*. *vivax* infection display increased cytotoxic function compared to other CD8^+^ T cells. Hence their function might be to kill parasitized reticulocytes through MHC-I dependent and perforin-mediated mechanisms. This proposal is further supported by the enhanced expression of cytotoxic molecules observed in CD38^+^ CD8^+^ T cells circulating during *P*. *vivax* compared to *P*. *falciparum* infection. In contrast, we could not identify an expansion of cytotoxic CD8^+^ T cell population following *P*. *falciparum* infection of malaria-naïve volunteers.

These findings have important implications in regard to *P*. *vivax* vaccine development. Indeed, most efforts so far have been directed toward the direct translation of the findings associated with *P*. *falciparum* vaccine development to *P*. *vivax* [20] (e.g. the use of ortholog antigens that were shown to be protective against *P*. *falciparum*). Here we show that *P*. *falciparum* and *P*. *vivax* elicit qualitatively different immune responses and are likely to require distinct vaccine-induced immune responses for protection. Thus, the immunization strategy may need to be adapted for each *Plasmodium* species to mount optimal protective immune responses, and the design of a universal vaccine conferring protection against multiple *Plasmodium* species might be conceptually more challenging than initially thought.

In conclusion, in this study we report that primary exposure of humans to different *Plasmodium* species elicited qualitatively distinct immune responses: *P*. *falciparum* infection generated changes in CD4^+^ T cells whereas *P*. *vivax* preferentially activated a subset of CD8^+^ T cells expressing the activation marker CD38 and associated with a cytotoxic function. The specific expansion of CD38^+^ CD8^+^ T cells following *P*. *vivax* infection might be due to the preference for *P*. *vivax* merozoites to infect reticulocytes which can activate CD8^+^ T cells through MHC-I dependent mechanisms. Overall our data are consistent with the proposal that protective immune responses to *Plasmodium* are species-dependent. These findings have important implications for malaria vaccine development strategies.

## Supporting Information

S1 Fig Parasite burden following experimental *P*. *vivax* blood-stage infection does not correlate with the expansion of CD38^+^ CD8^+^ T cells. (TIF)

S1 Table Demographics of *P*. *falciparum* and *P*. *vivax* infected volunteers (DOCX)

S2 Table Adverse events that occurred in *P*. *falciparum* and *P*. *vivax* infected volunteers until anti-malarial drug treatment (DOCX)
