# Supplementary figures and images for: Plasmodium vivax but Not Plasmodium falciparum Blood-Stage Infection in Humans Is Associated with the Expansion of a CD8+ T Cell Population with Cytotoxic Potential
Source: PLoS Negl Trop Dis. 2016 Dec 8;10(12):e0005031. doi: 10.1371/journal.pntd.0005031 (PMC5145136; doi:10.1371/journal.pntd.0005031)

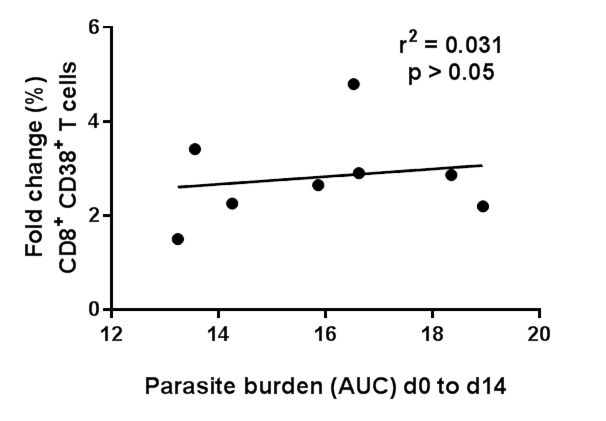

Supplement: S1 Fig — (TIF) [file pntd.0005031.s001.tif]
