# Supplementary material for: Plasmodium vivax but Not Plasmodium falciparum Blood-Stage Infection in Humans Is Associated with the Expansion of a CD8+ T Cell Population with Cytotoxic Potential
Source: PLoS Negl Trop Dis. 2016 Dec 8;10(12):e0005031. doi: 10.1371/journal.pntd.0005031 (PMC5145136; doi:10.1371/journal.pntd.0005031)
Supplement: S1 Table — (DOCX) [file pntd.0005031.s002.docx]

**S1 Table**

|  | ***P. falciparum* infected volunteers** | ***P. vivax* infected volunteers** |
| --- | --- | --- |
| **Age (years)^a^** | 24 [19-41] | 29 [22-34] |
| **Gender (Female/Male)** | 13/6 (68%) | 5/3 (63%) |
| **BMI (kg/m^2^)^a^** | 22.7 [18.3-26.1] | 25.5 [21.8-29.6] |
| **Ethnicity (Caucasian/Asian)** | 18/1 (95%) | 7/1 (88%) |
| ^a^Average[Min-Max] |  |  |
