# Supplementary material for: Plasmodium vivax but Not Plasmodium falciparum Blood-Stage Infection in Humans Is Associated with the Expansion of a CD8+ T Cell Population with Cytotoxic Potential
Source: PLoS Negl Trop Dis. 2016 Dec 8;10(12):e0005031. doi: 10.1371/journal.pntd.0005031 (PMC5145136; doi:10.1371/journal.pntd.0005031)
Supplement: S2 Table — (DOCX) [file pntd.0005031.s003.docx]

**S2 Table**

|  | ***P. falciparum* infected volunteers** | | ***P. vivax* infected volunteers** | |
| --- | --- | --- | --- | --- |
| **Adverse Event** | Nb of events | Nb of subjects affected | Nb of events | Nb of subjects affected |
| AST/ALT elevation | 2 | 2 | 0 | 0 |
| Anemia | 1 | 1 | 0 | 0 |
| Arthralgia | 0 | 0 | 4 | 4 |
| Body ache | 0 | 0 | 2 | 2 |
| Chills | 0 | 0 | 2 | 2 |
| CNS disturbance | 1 | 1 | 0 | 0 |
| Diarrhoea | 0 | 0 | 1 | 1 |
| Dizziness | 0 | 0 | 1 | 1 |
| Extreme thirst | 0 | 0 | 1 | 1 |
| Fatigue | 1 | 1 | 2 | 2 |
| Feeling clammy | 0 | 0 | 1 | 1 |
| Fever | 1 | 1 | 6 | 2 |
| Flu-like symptoms | 0 | 0 | 4 | 4 |
| Headache | 7 | 4 | 9 | 6 |
| Insomnia | 0 | 0 | 1 | 1 |
| Lethargy/tiredness | 2 | 1 | 4 | 2 |
| Leukopenia | 2 | 2 | 2 | 2 |
| Light headed | 0 | 0 | 1 | 1 |
| Loss of appetite | 0 | 0 | 1 | 1 |
| Malaise | 0 | 0 | 1 | 1 |
| Myalgia | 1 | 1 | 2 | 2 |
| Nausea | 1 | 1 | 4 | 4 |
| Neutropenia | 1 | 1 | 0 | 0 |
| Paraesthesia | 1 | 1 | 0 | 0 |
| Sensitive skin | 0 | 0 | 1 | 1 |
| Stomach ache | 0 | 0 | 1 | 1 |
| Sweats | 0 | 0 | 2 | 2 |
| Thrombocytopenia | 0 | 0 | 1 | 1 |
| Tingling fingers | 0 | 0 | 1 | 1 |
| URTI symptoms | 2 | 2 | 1 | 1 |
| **Total** | 23 | 11/19 (58%) | 56 | 8/8 (100%) |
